# Frontal Sinus Adenocarcinoma: A Rare Case of Diagnostic Re-evaluation From Inverted Papilloma

**DOI:** 10.7759/cureus.67878

**Published:** 2024-08-26

**Authors:** Samuel Chu, Salina Husain

**Affiliations:** 1 Department of Otorhinolaryngology-Head and Neck Surgery, Hospital Ampang, Ampang, MYS; 2 Department of Otorhinolaryngology-Head and Neck Surgery, Faculty of Medicine, Universiti Kebangsaan Malaysia, Kuala Lumpur, MYS

**Keywords:** sinonasal adenocarcinoma, otorhinolaryngology, rhinology, inverted papilloma, frontal sinus adenocarcinoma

## Abstract

Adenocarcinoma of the frontal sinus is extremely rare. We present a primary frontal sinus adenocarcinoma masquerading as an inverted papilloma (IP). Here, we reviewed various clinical presentations, investigations, and management of frontal sinus adenocarcinoma. A 48-year-old male presented with nasal bridge swelling one month following endoscopic sinus surgery for frontal sinus inverted papilloma. Progressively enlarging swelling with persistent pressure symptoms drew doubts regarding previously proven diagnosis. Imaging studies put us at the management crossroads of malignancy versus infection (osteomyelitis). The complexity of this case prompted a multidisciplinary team approach, eventually leading to a revision surgery for re-evaluation. Re-excision of the frontal sinus tumor was later proven to be adenocarcinoma of the frontal sinus. This case underscores the importance of thorough follow-up and investigation in patients presenting with recurrent or persistent symptoms following sinus surgery. This case highlighted the need for a high index of suspicion and comprehensive diagnostic workup.

## Introduction

Sinonasal malignancies are rare and often aggressive neoplasms arising from the nasal and paranasal sinuses [1]. Sinonasal malignancies display considerable histopathological heterogeneity, and adenocarcinoma represents the third most common sinonasal malignancy after squamous cell carcinoma and adenoid cystic carcinoma [2]. Adenocarcinomas predominantly affect males and most frequently occur in the ethmoid sinuses, but they can also originate from other sites of the nasal cavity. Primary or isolated carcinoma of the frontal sinus is extremely rare; it is estimated to account for 0.3%-1% of all paranasal sinus carcinomas and 0.009%-0.03% of head and neck cancers [3].

Inverted papilloma (IP) is a benign tumor found in the sinonasal tract [4]. Embryonically derived from the ectodermal Schneiderian membrane of the nasal cavity, IP may extend into the paranasal sinuses as they grow. In a review by Kim et al. [4] of patients diagnosed with IP, 7% of patients had associated malignancy. The same study found that tumors originating in the frontal sinus or frontoethmoidal recess tend to be associated with carcinoma.

We describe a rare case of primary adenocarcinoma of the frontal sinus masquerading as IP.

## Case presentation

A 48-year-old male military officer presented with swelling over the nasal bridge one month after undergoing endoscopic sinus surgery and a modified Lathrop procedure for a frontal sinus inverted papilloma. The patient, a known case of hypertension and a non-smoker, reported being reasonably well for one month postoperatively until he noticed the swelling, which gradually increased in size and was associated with a sensation of pressure described over the forehead and maxillary sinus region. The patient denied experiencing nasal obstruction, foul-smelling nasal discharge, smell disturbances, epistaxis, and postnasal drips. He also did not complain of any orbital or ontological symptoms.

**Figure 1:**
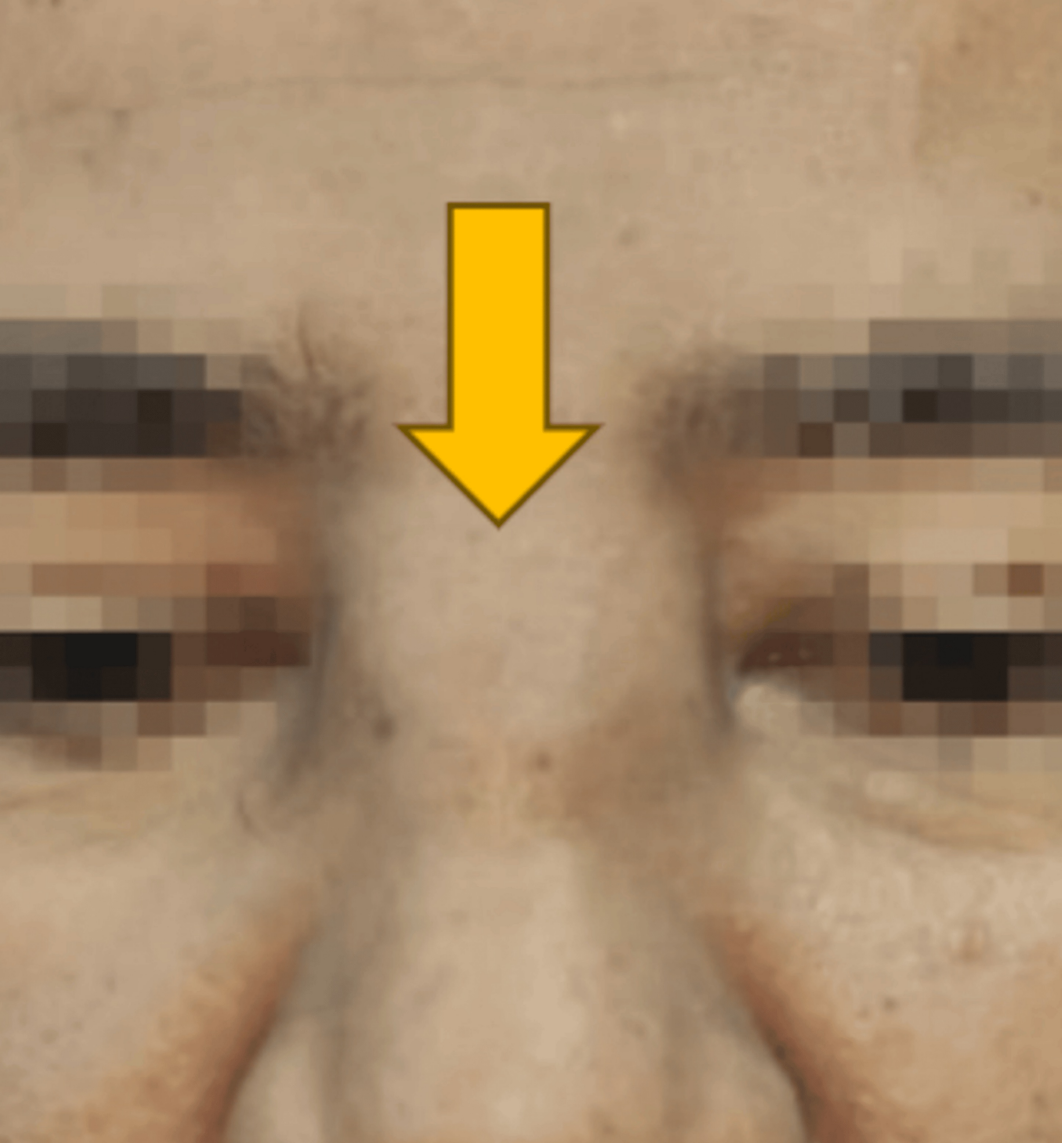
Swelling between both medial canthi, extending from the nasion superiorly down to the rhinion inferiorly

Physical examination revealed a swelling measuring approximately 3×3 cm over the region between both medial canthi, extending from the nasion superiorly to the rhinion inferiorly. The swelling was tender on palpation, and a saddle nose deformity was present (Figure 1). There were no eye signs, and extraocular movements were full. Nasal endoscopy showed evidence of bilateral full functional endoscopic sinus surgery (FESS) and modified Lothrop procedure with a mass over the right frontal recess. The frontal sinus was stenosed. Reactionary polypoidal mucosa was noted over the right ethmoidal sinus. The neck examination was unremarkable.

**Figure 2:**
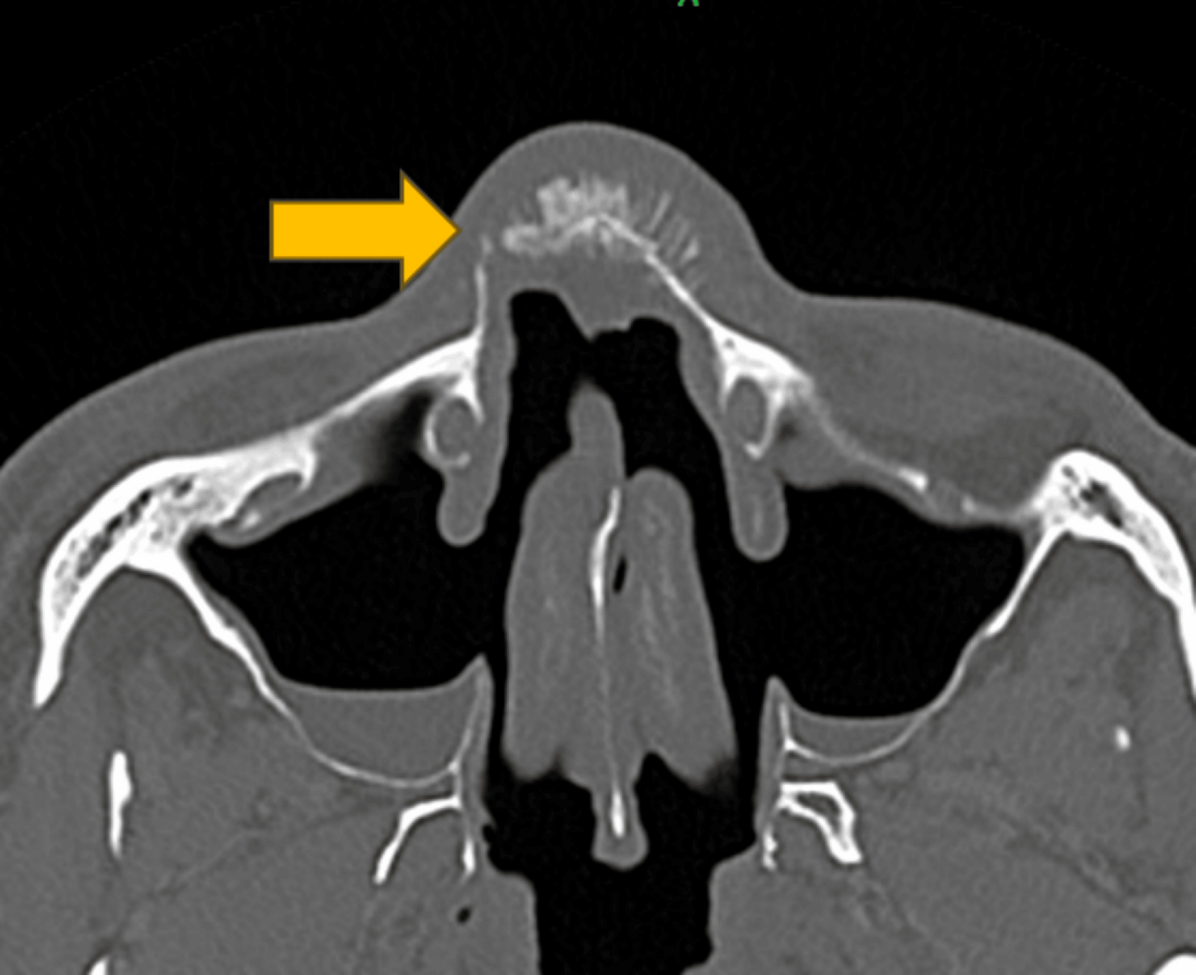
Aggressive sunburst periosteal reaction of nasal bone with remarkable soft tissue thickening, evidence of prior maxillary sinus surgery (axial plane, bone window)

**Figure 3:**
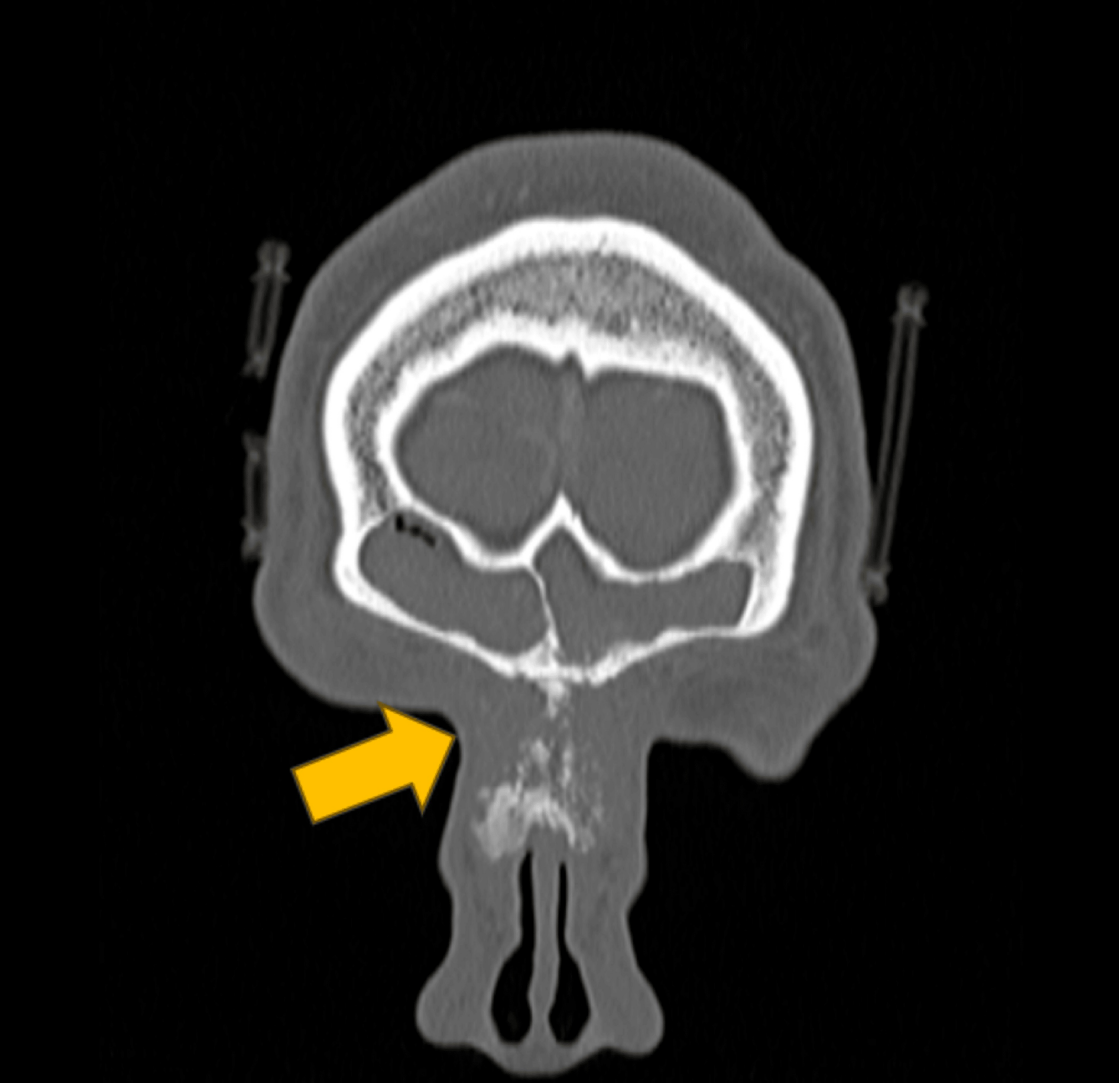
Homogenous enhancing soft tissue density within the left frontal sinus and aggressive sunburst periosteal reaction of nasal bone seen on CT scan (coronal plane, bone window) CT: computed tomography

The diagnosis of inverted papilloma was confirmed by histopathological examination (HPE) following the initial surgery. However, the patient's current presentation raised concerns of recurrent or residual disease. The differential diagnosis of infective etiology was considered, and the patient was covered with intravenous (IV) antibiotics (IV Augmentin 1.2 g TDS and IV Flagyl 500 mg TDS). A computed tomography (CT) scan of the paranasal sinuses, brain, and neck revealed a homogenous enhancing soft tissue density within the left frontal sinus and aggressive sunburst periosteal reaction of the nasal bone suggestive of frontal bone osteomyelitis (Figure 2 and Figure 3). Following the CT scan, the antibiotics regime was escalated to IV ciprofloxacin 400 mg TDS.

A multidisciplinary team approach was employed, incorporating expert consults from the radiology and infectious disease teams. Discussion with the radiologist yielded that the sunburst pattern over the nasal bridge is a feature of aggressive periosteal reaction suggestive of osteosarcoma or severe osteomyelitis. The etiology of the patient's illness was unlikely infective in nature due to unraised infection markers (C-reactive protein: 0.17 mg/L, total white cell count: 7.5×10^9^/L).

Given the diagnosis dilemma, a revision Draf III procedure and biopsy were planned. Intraoperative findings included obliterated frontal sinus with suspicious residual tissue, polypoidal mucosa over the posterior table of the right frontal sinus with thick mucoid discharge, and bony swelling at the nasal bridge with brittle body fragments. Biopsy was taken from multiple sites. HPE showing malignant cells confirmed the diagnosis of adenocarcinoma (from frontal sinus mass and nasal bone).

The patient recovered well post-surgery. He was referred to an oncologist for the continuation of care and development of a post-surgery treatment plan.

## Discussion

Frontal sinus malignancies are extremely rare [5]. Literature on primary frontal sinus malignancies is scarce. Wide histological heterogeneity and treatment strategies are described. A 40-year database-based study by Bhojwani et al. [6] reported that 171 cases of malignancies involving frontal sinuses had poor prognosis, with an average five-year survival rate of 44.2%. The study also found that the five-year survival rate was highest for mature B-cell non-Hodgkin's lymphomas (72.3%) and lowest for adenocarcinomas (15.4%) [6]. Common manifestations include forehead swelling, frontal headache, and signs of orbital invasion presenting with proptosis and diplopia. Symptoms are generally associated with an extension of disease beyond frontal sinus boundaries to adjacent regions such as the intracranial compartment and orbit. Patients often present late when the tumor is large enough to manifest physical symptoms.

In view of the scarcity of frontal sinus malignancies, this sinus is not considered a "primary site" in the American Joint Committee on Cancer (AJCC) [7]. This staging system mentioned the nasal cavity, maxillary, and ethmoid sinuses as the primary site of cancer, with frontal sinus only in cases of secondary involvement.

Inverted papilloma is a benign but locally aggressive sinonasal neoplasm. Such lesions can be associated with squamous cell carcinoma in approximately 7%-16% of cases, either synchronously or metachronously [4]. Differentiating benign IP and IP associated with malignancy can be challenging clinically as patients can present with almost identical clinical symptoms [8]. Sometimes, diagnosis can be missed by biopsy because tissue containing malignant cells is not always obtained. We have not come across literature describing IP masquerading as an adenocarcinoma.

Diagnosis is aided with imaging such as CT and magnetic resonance imaging (MRI), which is done preceding biopsy [5]. CT and MRI are useful tools for the assessment of tumor size, extent, and degree of invasion [9]. CT is most commonly used as it is widely available, has lower cost, and offers greater anatomical detail. CT is superior to MRI in delineating calcification and evaluating patterns of bone invasion. Intralesional calcifications are observed in some cases of sinonasal adenocarcinoma. The characteristic of bone invasion helps to predict the histology of malignancy. Whenever feasible, a biopsy should be done via a minimally invasive approach (i.e., endoscopic transnasal approach). Histopathological examination is essential for determining the histology type, which will guide subsequent multidisciplinary treatment strategies.

Among rhinologists, the frontal sinus is deemed to be one of the more challenging areas to approach surgically due to poor visualization of the frontal recess [10]. An external approach (i.e., transcranial/transfacial) might be needed to manage specific situations where a transnasal endoscopic approach is not possible. The goal of surgery is to attain free-margin resection. The involvement of margins has shown to be a poor prognostic factor. In cases of the management of benign and malignant masses of the frontal sinus, the Draf III procedure is performed to provide adequate surgical access for tumor excision. Draf III is the most extensive of the Draf procedures and aims at creating a common frontal sinus cavity. The Draf III limits of resection are the external periosteum of the frontal process of the maxilla anteriorly, the first olfactory fibers posteriorly, and the lamina papyracea laterally.

Despite being an aggressive malignancy, sinonasal adenocarcinoma is potentially curable [2]. The long-term survival rate is best achieved with early diagnosis and adequate multidisciplinary team management. The most important factor influencing the long-term survival of patients with adenocarcinoma is radical surgical resection of the tumor (i.e., margin-free clearance). At the moment, there is no clear literature that supports the use of adjuvant radiotherapy as compared to surgery alone.

## Conclusions

This case underscores the importance of thorough follow-up and investigation in patients presenting with recurrent or persistent symptoms following sinus surgery. The initial diagnosis of inverted papilloma, which was HPE-proven, masked an underlying adenocarcinoma of the frontal sinus, which is extremely rare. This case highlighted the need for a high index of suspicion and comprehensive diagnostic workup.

The role of detailed imaging studies is also highlighted. The presence of a sunburst pattern and aggressive periosteal reaction in the absence of infective biochemical markers were key findings that guided further investigation and eventual diagnosis of an underlying malignancy. The benefits of a multidisciplinary approach, involving otolaryngologists, radiologists, physicians, pathologists, and oncologists, are further emphasized in the management of complex cases.
